# Emotional bias training as a treatment for anxiety and depression: evidence from experimental medicine studies in healthy and medicated samples

**DOI:** 10.1017/S0033291721002014

**Published:** 2021-05-31

**Authors:** Steph Suddell, Maren Müller-Glodde, Jim Lumsden, Chung Yen Looi, Kiri Granger, Jennifer H. Barnett, Oliver J. Robinson, Marcus R. Munafò, Ian S. Penton-Voak

**Affiliations:** 1 School of Psychological Science, University of Bristol, 12a Priory Road, Bristol BS8 1TU, UK; 2 NIHR Bristol Biomedical Research Centre, University Hospitals Bristol and Weston NHS Foundation Trust, University of Bristol, Oakfield House, Oakfield Grove, Bristol BS8 2BN, UK; 3 Medical Research Council (MRC) Integrative Epidemiology Unit at the University of Bristol, Oakfield House, Oakfield Grove, Bristol BS8 2BN, UK; 4 Cambridge Cognition Ltd., Tunbridge Court, Tunbridge Lane, Bottisham, Cambridge CB25 9TU, UK; 5 Department of Psychiatry, University of Cambridge, Herchel Smith Building, Robinson Way, Cambridge CB2 0SZ, UK; 6 Institute of Cognitive Neuroscience, University College London, Alexandra House, 17-19 Queen Square, Bloomsbury, London WC1N 3AR, UK

**Keywords:** Anxiety, cognitive training, depression, digital intervention, emotional processing

## Abstract

**Background:**

Anxiety and depression are leading causes of disability worldwide, yet individuals are often unable to access appropriate treatment. There is a need to develop effective interventions that can be delivered remotely. Previous research has suggested that emotional processing biases are a potential target for intervention, and these may be altered through brief training programs.

**Methods:**

We report two experimental medicine studies of emotional bias training in two samples: individuals from the general population (*n* = 522) and individuals currently taking antidepressants to treat anxiety or depression (*n* = 212). Participants, recruited online, completed four sessions of EBT from their own home. Mental health and cognitive functioning outcomes were assessed at baseline, immediately post-training, and at 2-week follow-up.

**Results:**

In both studies, our intervention successfully trained participants to perceive ambiguous social information more positively. This persisted at a 2-week follow-up. There was no clear evidence that this change in emotional processing transferred to improvements in symptoms in the primary analyses. However, in both studies, there was weak evidence for improved quality of life following EBT amongst individuals with more depressive symptoms at baseline. No clear evidence of transfer effects was observed for self-reported daily stress, anhedonia or depressive symptoms. Exploratory analyses suggested that younger participants reported greater treatment gains.

**Conclusions:**

These studies demonstrate the effectiveness of delivering a multi-session online training program to promote lasting cognitive changes. Given the inconsistent evidence for transfer effects, EBT requires further development before it can be considered as a treatment for anxiety and depression.

Depression and anxiety are leading causes of disability worldwide (World Health Organization, 2017). Current treatment strategies include antidepressant medications and psychotherapy. While these can be effective, they are often difficult to access, financially costly, and associated with side effects. A substantial proportion of patients will not respond to their first-line treatment (Rush, 2007). There is an urgent need to address this treatment gap by developing effective treatments that can be delivered rapidly and remotely. Digital health technologies are being increasingly explored as a method to deliver both standalone and adjunct therapies for mental health disorders (Hollis et al., 2015). These therapies must be validated by real-world studies to ensure they are safe, evidence-based, and effective.

A potential target for intervention is biased emotional processing (Penton-Voak, Munafò, & Looi, 2017). Depressed and anxious individuals display negative biases when processing ambiguous social cues (Beevers, Wells, Ellis, & Fischer, 2009; Bourke, Douglas, & Porter, 2010; Hayes & Hirsch, 2007). For example, when viewing ambiguous facial expressions, depressed individuals may be more likely to interpret them as showing a sad or hostile emotion, rather than a positive emotion. This tendency may play a causal role in the development of low mood, as individuals receive undue negative feedback from their environment and subsequently respond maladaptively, worsening their social experience further. Current pharmacological treatments, such as selective serotonin reuptake inhibitors (SSRIs), appear to alter emotional processing early in the treatment course, and these changes are associated with symptom remittance at a later time-point (Godlewska, Browning, Norbury, Cowen, & Harmer, 2016; Harmer, Goodwin, & Cowen, 2009). In neurocognitive models, this reduction of negative processing allows individuals to experience the world more positively, which in time leads to improvements in symptoms and cognitive functioning (Pringle, Browning, Cowen, & Harmer, 2011; Warren, Pringle, & Harmer, 2015). This reasoning has led to the development of interventions that modify emotional processing through non-invasive behavioural, rather than pharmacological, methods (Hallion & Ruscio, 2011; Menne-Lothmann et al., 2014), with the hope that changes to this cognitive target lead to symptom remittance.

We have developed an intervention that targets negative processing biases through emotional bias training (EBT). EBT uses feedback to train participants to interpret ambiguous facial expressions as displaying a positive rather than a negative emotion, with the aim of inducing a positivity bias. It is theorised that this positivity bias will have beneficial downstream effects on the participants’ interpretation of their wider social experiences, which are preferable to those produced by more negative interpretations. In short, this is achieved by asking participants to categorise a continuum of ambiguous faces, ranging from very happy to very sad, in order to determine their emotional ‘balance point’ (the point where they perceive a switch between ‘happy’ and ‘sad’). Participants are then trained to categorise more of the ambiguous faces as happy (i.e. trained to a higher balance point). This approach is effective in modifying emotional processing in a range of populations, including individuals suffering from emotional and conduct disorders (Penton-Voak, Bate, Lewis, & Munafò, 2012, 2013; Rawdon et al., 2018). It has also shown promise as a tool to improve mood, with some studies reporting increases in positive affect, reduced self-reported anger and improved stress resilience (Penton-Voak et al., 2012, 2013; Peters et al., 2017). However, the effectiveness of EBT is yet to be studied when delivered in a real-world setting.

A recent neuroimaging study found that EBT alters emotional processing by increasing neural activation in response to happy facial expressions (Penton-Voak et al., 2020). After five sessions, participants showed increased activity in the medial prefrontal cortex and bilateral amygdala when viewing a novel set of happy faces. These brain areas are implicated in emotion regulation and mood disorders (Disner, Beevers, Haigh, & Beck, 2011), and related changes in activity are identified during SSRI treatment. For example, depressed individuals in their 1st week of SSRI treatment have a reduction in neural activity in the amygdala when viewing fearful (*v*. happy) faces (Godlewska, Norbury, Selvaraj, Cowen, & Harmer, 2012, 2016). Importantly, these changes preceded the remittance of symptoms. While the exact neural mechanism of EBT requires further investigation, it is promising that our digital intervention appears to target similar pathways to current pharmacological treatments for anxiety and depression. One aim of the current research is to evaluate whether administering EBT as an adjunct therapy to SSRIs has the potential to offer additional benefit.

Here we describe two pre-registered studies evaluating EBT as a digital intervention for anxiety and depression. In both studies, participants were recruited and completed all study sessions online from their own homes, providing a test of the intervention in a ‘real world’ setting in which digital therapeutics may be delivered. The EBT procedure closely matched those in previous laboratory-based studies (Penton-Voak et al., 2020), and was delivered over four sessions, which has been shown to produce enduring changes on the emotional bias task (e.g. at 2-week follow-up; Rawdon et al., 2018). Participants returned for a 2-week follow-up, which allowed us to assess the effects of training after participants had a reasonable opportunity to engage in social interactions. We assessed several mental health and functional outcomes known to be associated with anxiety and depression.

In study 1, we examined the effectiveness of EBT in a sample of healthy volunteers to test the feasibility of conducting the EBT trial remotely and to explore Peters et al.’s (2017) finding that EBT may lead to a reduction in daily stress in healthy participants. Mental health and functional outcomes were assessed at baseline, immediately post-training, and at a 2-week follow-up. We hypothesised that four sessions of EBT would induce a positivity bias in the recognition of emotional facial expressions, as shown in previous laboratory-based research (Penton-Voak et al., 2012). We also hypothesised that this change in bias would transfer to decreased daily stress and greater motivation to seek reward (Peters et al., 2017). We also explored if EBT led to self-reported improvements in quality of life.

In study 2, we recruited volunteers who were currently taking SSRIs for anxiety or depression, to investigate the potential of EBT as an adjunct therapy. Based on study 1 findings, we hypothesised that an EBT induced positivity bias would transfer to an improvement in self-reported quality of life. In addition, we investigated transfer effects to other mental health and functional outcomes: daily stress, anhedonia, depressive symptoms, state anxiety and ‘feeling of treatment’ (the extent to which the participant felt their current treatment was working for them).

## Method

### Overview

Both studies were preregistered on the Open Science Framework (study 1: osf.io/qf28h/; study 2: osf.io/wuvjb/), where full methods can be found. Ethics approval was granted by the Faculty of Science Human Research Ethics Committee of the University of Bristol (study 1: 53001; study 2: 55441).

### Emotional bias training

Our training paradigm modifies participants’ perception of emotional facial expressions, by encouraging them to perceive more ambiguous facial expressions as positive (happy) rather than negative (sad) emotion. Each training session consists of three blocks: baseline, training, and test. In all three blocks, participants respond to a series of faces, indicating whether they perceive them to be ‘happy’ or ‘sad’. Each face stimulus is from a continuum of 15 morphed faces, that range in intensity from unambiguously happy (face 1) to unambiguously sad (face 15) (see original paper for further details; Penton-Voak et al., 2012)

In the baseline block, participants respond to 45 trials (all 15 faces, presented 3 times) and receive no feedback regarding their categorisation of the faces. This determines the balance point (i.e. face number) at which participants shifted from perceiving happiness to perceiving sadness. This is calculated by the number of faces judged as ‘happy’ divided by a number of trials, multiplied by 15. In the training block, participants receive feedback on each trial, indicating whether their categorisation was ‘Correct’ or ‘Incorrect’ (Online Supplementary Fig. S1). Feedback is tailored to each participant, based on their baseline balance point. For participants receiving active training, feedback promotes recognition of two more faces (along the 15-point continuum) as ‘happy’. In the sham condition, feedback is given in line with their baseline balance point, meaning no training is administered. The final (test) block is identical to the baseline block, in order to measure any immediate changes in the participants’ balance point after training. An entire training session takes approximately 8.5 min to complete.

### Participants

Study 1 recruited healthy participants via Prolific (www.prolific.co), an online recruitment platform. Eligible participants were above 18 years old, fluent in English, and had normal or corrected-to-normal colour vision. Participants were ineligible if they were taking anti-depressants, anxiolytics or anti-psychotics, or had consumed alcohol within the last 12 h. Participants who completed all sessions were reimbursed £10, plus any additional monetary reward earned during the study (see study 1 Measures).

Study 2 participants, also recruited via Prolific, were currently taking SSRIs (either citalopram, escitalopram, fluoxetine, paroxetine, sertraline or vilazodone) to manage anxiety and/or depression. Eligible participants were above 18 years old, had English as a first language (or equivalent) and had a normal or corrected colour vision. Participants were ineligible if they had participated in study 1, or consumed alcohol 12 h before the study session. Participants who completed all sessions were reimbursed £10.

In both studies, participants accessed information sheets (hosted on Prolific) prior to their participation and gave their informed consent (via a checkbox) upon entering the study.

### Design

Both studies used a single-blind randomised controlled design. As this study was conducted remotely, experimenters were unable to bias participants’ performance. Participants were randomly assigned (by random number generator) to active or sham EBT. All participants were required to complete four training sessions within 10 days, and a follow-up session approximately 2 weeks after their final training. Mental health and functional outcomes were assessed at baseline (start of session 1), post-training (end of session 4), and follow-up (end of session 5).

In study 2, we replaced the EBT test block of sessions 1, 2 and 3 with a second training block, and added an additional training block to session 4. This doubled the number of training blocks (from 4 to 8) while maintaining a similar study duration.

### Study 1 measures

The primary outcome of study 1 was self-reported daily stress, immediately post-training. In line with previous work (Peters et al., 2017), we assessed daily stress through average impact rating on the daily stress inventory (DSI-AIR; Brantley, Waggoner, Jones, & Rappaport, 1987). This measure captures participants’ self-reported stress responses in the past 24 h, to a range of commonly occurring stressors. Secondary measures included quality of life and effort for reward. Quality of life was measured with the Quality of Life Enjoyment and Satisfaction questionnaire (QLES, short-form version; Endicott, Nee, Harrison, & Blumenthal, 1993) and a percentage of maximum score (out of 56) was calculated for each participant. Effort for reward was assessed through the Effort Expenditure for Reward Task (EEfRT; Treadway, Buckholtz, Schwartzman, Lambert, & Zald, 2009), an objective measure of anhedonia, which required participants to press sequences of keys in return for differing monetary rewards. Participants were given the choice between ‘easy’ and ‘hard’ trials and scored on the proportion of hard trials chosen. See online Supplementary Materials for full description.

Depression and anxiety (state and trait) were assessed via the PHQ-9 (Spitzer, Kroenke, & Williams, 1999) and STAI (Y1 and Y2 forms; Spielberger, Gorsuch, Lushene, Vagg, & Jacobs, 1983), respectively. The suicide ideation question was removed from the PHQ-9 on the request of the ethics committee. Other measures (not discussed here, available in our open datasets) included the Immediate Mood Scaler (Nahum et al., 2017) and a brief survey of participants’ social experiences in the past 24 h, at each session. These were collected alongside information regarding the usability of the intervention for task development purposes, should the intervention be successful.

### Study 2 measures

The primary outcome of study 2 was quality of life (QLES; percentage maximum score) as study 1 suggested EBT may lead to an improvement in QLES amongst individuals with depressive symptoms. Secondary outcomes included daily stress (DSI-AIR), depressive symptoms (PHQ-9), state anxiety (STAI-Y1), and anhedonia. Anhedonia was assessed using the Snaith-Hamilton Pleasure Scale (SHAPS; Snaith et al., 1995), a self-report measure of anhedonia, rather than the EEfRT used in study 1.

In study 2, participants provided information regarding the duration of SSRI treatment and whether they were currently receiving talking therapy, either online or in-person. Participants also reported their ‘feeling of treatment’ (whether they felt improved by their current treatment for anxiety or depression) at baseline, post-training and follow-up. This was a 5-point scale ranging from ‘much worse’ to ‘much better’.

### Sample size determination

Study 1 sample size was determined based on the previously identified effect size of group difference in daily stress (DSI-AIR) following EBT (*d* = 0.35) (Peters et al., 2017). A sample of 586 participants would allow us to detect this effect with 95% power and an alpha level of 1%, and a more conservative effect of *d* = 0.23 with 79% power at an alpha level of 5%. A total of 891 participants signed up to the study. Of these 874 participants accessed the study website and 527 provided complete datasets. These datasets were screened for outliers and inappropriate responding (see protocol for full details), resulting in a final sample of 522 participants (see online Supplementary Fig. 2).

Study 2 sample size was determined based on a small to medium effect size of *d* = 0.30 for quality of life (QLES), equivalent to 4.2 points on this measure (expressed as a percentage maximum score). We originally calculated that a sample size of 278 would allow us to detect this effect with 80% power and an alpha level of 5%. However, this was erroneously based on a one-sided test in our pre-registered study protocol. A total of 500 participants were recruited for study 2, 233 of which provided complete datasets. The final sample for study 2 consisted of 212 participants after the removal of 21 outliers as per protocol (see online Supplementary Fig. 3). This sample size provided 58% power at an alpha level of 5% to detect an effect size of *d* = 0.3, and 80% power to detect an effect size of *d* = 0.39 (equivalent to 5.5 points on the QLES).

Sample size calculations were conducted in G*Power (Faul, Erdfelder, Buchner, & Lang, 2009).

### Statistical analyses

Statistical analyses were conducted in RStudio (RStudio Team, 2015). For each study, we conducted a series of linear regression analyses to investigate the effect of condition (active/sham EBT) on mental health and cognitive outcomes post-training. Analyses were adjusted for age, sex, and baseline score of the respective measure. These models were repeated for study outcomes at follow-up (Session 5) to investigate the longer-term effects of EBT.

In study 1, the second set of models additionally adjusted for baseline depression (PHQ-9) and state anxiety (STAI-Y1) scores. We also investigated the effects of training in subgroup analyses stratifying by baseline depression and anxiety. For the depression analysis, participants were split by PHQ-9 score (>4 indicating signs of at least mild depression; Kroenke, Spitzer, & Williams, 2001). For anxiety, participants were median split into high and low trait anxiety by STAI-Y2 score (high anxiety >44).

In study 2, an additional set of models adjusted for length of SSRI use and whether the participant was receiving talking therapy (dummy coded as yes/no). Secondary analyses investigating the interaction of training with baseline mental health were then conducted as described for study 1. For the depression analysis, the sample was median split by PHQ-9 (>12). For anxiety, participants were median split by trait anxiety (STAI-Y2 >58).

## Results

### Participant characteristics

Baseline characteristics of both studies can be seen in Table 1. Study 1 recruited a sample of 522 adult participants who were not currently taking antidepressants, anxiolytics or antipsychotics. Of the 522 participants, 54% were female (sham condition: 45%, active condition: 61%).

Study 2 recruited 212 adult participants who reported that they were currently taking an SSRI to treat anxiety or depression (Citalopram: 26.4%, Escitalopram 3.8%, Fluoxetine: 21.7%, Paroxetine: 2.8%, Sertraline: 45.3%; average duration of 50 months). Of the participants, 82% were female (sham condition: 85%, active condition: 79%). The self-reported mood was generally low, with the average PHQ-9 score indicating moderate depression (Kroenke et al., 2001). 22% of participants reported currently receiving talking therapy.

### Correlations with baseline balance point

At baseline in study 1 (healthy participants), balance point was correlated with trait anxiety (STAI-Y2; *r*(520) = −0.10, *p* = 0.029), daily stress (DSI-AIR; *r*(520) = −0.10, *p* = 0.022) and quality of life (QLES; *r*(520) = 0.09, *p* = 0.034), indicating individuals with lower symptoms categorised more faces as ‘happy’. There was no clear evidence for correlations with depressive symptoms, state anxiety, or effort for reward (online Supplementary Table S6).

In study 2 (participants on medication), there was evidence for a small negative correlation with depressive symptoms (PHQ-9; *r*(210) = −0.15, *p* = 0.027), trait anxiety (STAI-Y2; *r*(210) = −0.12, *p* = 0.082), anhedonia (SHAPS; *r*(210) = −0.21, *p* = 0.002), and a positive correlation with the quality of life (QLES; *r*(210) = 0.16, *p* = 0.022). There was no clear evidence for a correlation with the feeling of treatment, daily stress, or state anxiety.

### Effectiveness of EBT on cognitive target

**Fig. 1:**
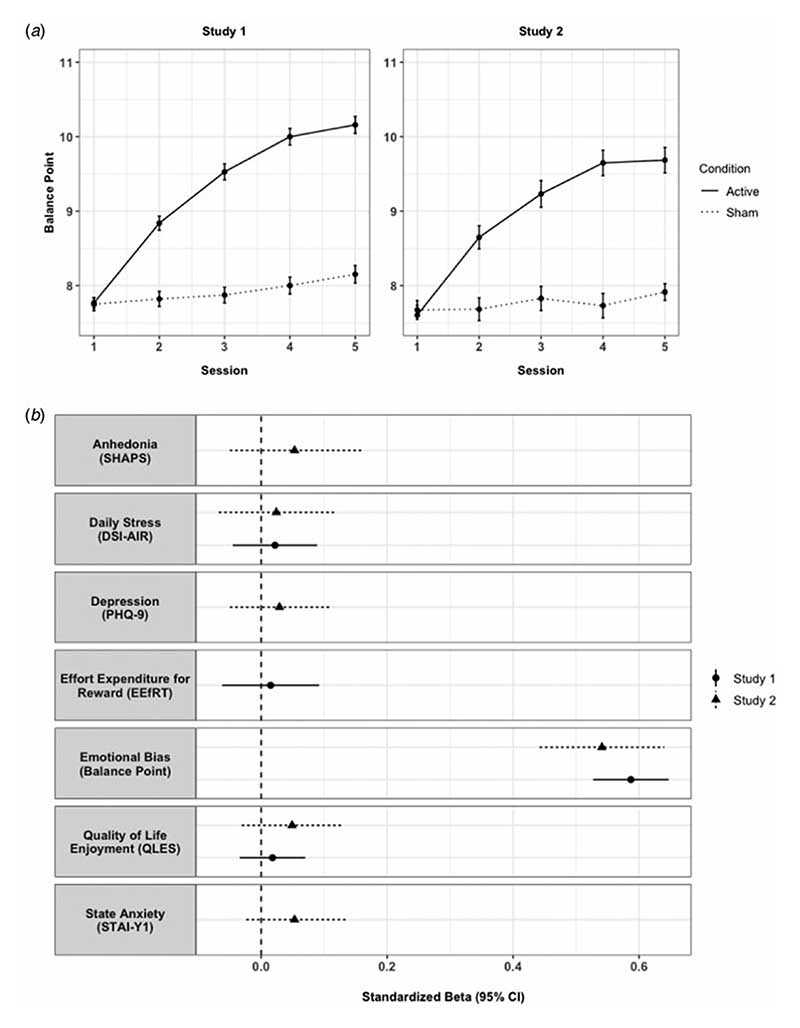
Effects of EBT across both study 1 (healthy participants) and study 2 (participants on medication). (*a*) Mean emotional balance point at each study session, per EBT condition. Error bars display standard error of the mean. Means are from the baseline (pre-training) block of that EBT session. Sessions 1–4 were completed within a 10-day period. Session 5 was completed approximately 2 weeks after Session 4. (*b*) Effect estimates of EBT condition on study outcomes. Standardized beta coefficients for the effect of condition on study outcomes immediately post-training (end of Session 4). Models were adjusted for age, sex, and baseline measure of the respective outcome. For ease of interpretation, the effect estimates for anhedonia, daily stress, depression, and state anxiety have been inverted (i.e. positive Betas now indicate improvement across all measures).

In both studies, EBT produced positive changes in participants’ emotional balance point (see Fig. 1, Panel A), meaning that participants classified more ambiguous faces as ‘happy’ following active training.

In study 1, participants in the active condition had a 2.6 point increase (95% CI 2.3–2.9, *p* < 0.001) in their balance point post-training and 2.0 point increase (95% CI 1.7–2.3, *p* < 0.001) at follow-up (fully adjusted models, Table 2), indicating that participants in the active branch continued to categorise more faces as ‘happy’ 2 weeks later.

There was some evidence for an interaction with baseline mental health (see Table 3 and online Supplementary Fig. S5), with EBT being less effective in individuals with depressive symptoms (*b* = −0.6, 95% CI −1.1 to −0.0, *p* = 0.033) and higher baseline trait anxiety (*b* = −0.7, 95% CI −1.3 to −0.2, *p* = 0.006). However, stratified analyses indicated that participants in the higher stratum on these measures still substantially increased their post-training balance point following EBT (with depressive symptoms: 2.3 points, 95% CI 2.0 to 2.7, *p* < 0.001; with high trait anxiety: 2.2 points, 95% CI 1.8 to 2.6, *p* < 0.001).

In study 2, the medicated sample, there was also strong evidence of EBT leading to an increase in balance point, both immediately post-training and at follow-up (Table 4). There was no clear evidence for an interaction between training condition and either baseline depression or trait anxiety on post-training balance point (depression: *b* = 0.2, 95% CI −0.7 to 1.0, *p* = 0.700; trait anxiety: *b* = −0.1, 95% CI −1.0 to 0.7, *p* = 0.779), suggesting that symptom severity had little impact on the effectiveness of training.

### Transfer effects in healthy participants (study 1)

In healthy participants, there was no clear evidence that active EBT had an effect on the primary outcome of daily stress (DSI-AIR; Table 2), nor secondary outcomes of effort for reward (EEfRT) and quality of life (QLES). However, all effect estimates were in the direction of improvement (see Fig. 1, Panel B for a plot of effect estimates across both studies).

There was little evidence for interactions between EBT condition and baseline mental health (level of trait anxiety and presence of depressive symptoms) on study outcomes (Table 3). There was some evidence for an interaction between depressive symptoms and EBT condition on post-training quality of life (QLES). Stratified analyses indicated that individuals with depressive symptoms reported increased quality of life following active EBT (*b* = 2.36, 95% CI −0.11 to 4.83, *p* = 0.061). This pattern did not remain at follow-up (see online Supplementary Table S1).

### Transfer effects in participants on medication (study 2)

Amongst individuals taking SSRIs, there was no clear evidence that active EBT had an effect on the quality of life immediately post-training (QLES; study 2 primary outcome). There was also no clear evidence for an effect at follow-up (unadjusted model: *b* = 2.59, 95% CI −0.36 to 5.54, *p* = 0.085; Table 4).

There was also no clear evidence for transfer effects of EBT when examining the secondary outcome measures, either immediately post-training or at follow-up (Table 4), and no evidence for interactions between EBT condition and baseline mental health (online Supplementary Tables S2 and S3). However, participants who received active EBT reported a greater feeling of treatment (i.e. that their current treatment was working for them; Table 4). This effect remained at follow-up.

### Interaction with Age

Exploratory inspection of the study 2 dataset suggested a potential interaction between the age of participants and condition, leading to unplanned posthoc analyses of age effects. We performed a median split by age (older >35 years) and ran linear regressions for all outcomes (adjusted for sex and baseline score of the outcome) in both age strata. There was evidence for condition and age interactions on post-training quality of life (QLES) and state-anxiety (STAI-Y1), and weaker evidence for interaction on post-training depressive symptoms (PHQ-9; see online Supplementary Table S4). Stratified analyses indicated that active EBT was associated with improvements on these measures in younger participants (quality of life: *b* = 4.54, 95% CI 0.91–8.17, *p* = 0.015; state anxiety: *b* = −5.13, 95% CI −8.07 to −2.20, *p* = 0.001, and depressive symptoms: *b* = −1.17, 95% CI −2.24 to −0.09, *p* = 0.034), but not older participants (all *p* > 0.10; online Supplementary Table S4). The effect of active EBT on quality of life and state anxiety persisted for younger participants at follow-up (quality of life: *b* = 6.17, 95% CI 2.10–10.24, *p* = 0.003; state anxiety: *b* = −4.19, 95% CI −8.03 to −0.35, *p* = 0.033). There was no clear evidence of age interactions for the remaining mental health outcomes; however, effect estimates were consistently larger for younger participants (and in the direction of improvement). For completeness, these analyses were repeated for the study 1 sample (older >32 years). No evidence of age effects was identified (online Supplementary Table S5).

## Discussion

Here we report two studies that investigated the effectiveness of EBT when remotely delivered as a digital therapeutic, in both healthy participants and individuals taking SSRIs for anxiety or depression. In both, EBT produced positive changes in facial emotion perception, with effects comparable to those found in laboratory-based studies (Penton-Voak et al., 2012, 2020). Previous experimental work has found that these changes can transfer to novel faces (Dalili, Schofield-Toloza, Munafò, & Penton-Voak, 2017), suggesting that a short course of EBT has the potential to influence participants’ wider social encounters. In study 1, there was some evidence these changes were larger in individuals with no self-reported depressive symptoms and lower levels of trait anxiety. This is perhaps unsurprising, given that depression has been found to be associated with cognitive impairment in attention and memory (Rock, Roiser, Riedel, & Blackwell, 2014). However, importantly, positive changes (which remained at 2-week follow-up) were identified across all samples, including individuals in study 2 who self-reported moderate to severe levels of depression. This result is encouraging for the use of digital therapeutics in emotional disorders more generally, demonstrating individuals with relatively high levels of symptoms can successfully adhere to and benefit from a multi-session online intervention. However, despite these positive changes in emotional bias, we found little evidence for transfer effects to mental health and functional outcomes, which suggests that EBT cannot currently be considered therapeutic.

In healthy participants (study 1), we did not find any effect of training on the primary outcome of self-reported daily stress, nor on the secondary outcomes of anhedonia (effort expenditure for reward) or quality of life. Previously, a laboratory-based study of EBT in healthy participants identified weak effects of EBT on the same measures of daily stress and anhedonia (Peters et al., 2017). The present study provides little evidence that changes in emotion perception are sufficient to produce significant changes in wellbeing for healthy individuals. As study 1 tested EBT in a healthy sample, it may be expected that participants would be unlikely to see substantial improvement in their existing levels of functioning. Stratified analyses provided some evidence for this, as effect estimates for changes in daily stress and quality of life were larger for participants with poorer mental health at baseline. In particular, there was some evidence that active EBT was associated with a greater quality of life when only examining individuals who reported depressive symptoms at baseline, despite these individuals showing a smaller training effect in terms of the emotional balance point. Tentatively, this may suggest that alterations in emotional processing have a greater impact in individuals with higher levels of symptoms. However, this pattern was not found at a 2-week follow-up.

In study 2, participants, who were taking SSRIs, reported moderate levels of depression at baseline (mean PHQ-9 score of 12). Previous research has suggested SSRIs may have a therapeutic effect by altering biased emotional processing (Warren et al., 2015). We sought to investigate whether the additional administration of EBT could provide further benefit. Our results provide little evidence for this, with positive changes in emotion perception not appearing to transfer to symptoms. However, once again, effect estimates were consistently in the direction of improvement.

Post-hoc analyses of study 2 indicated that active EBT had stronger therapeutic effects in younger participants (<35 years), finding positive changes in state anxiety, depressive symptoms and quality of life. This apparent interaction with participant age cannot be explained by the effectiveness of the training in terms of changes in emotion perception, which appeared equally effective for younger and older participants. Previous studies of EBT in younger participant groups, such as dysphoric undergraduates and adolescents with social anxiety (Penton-Voak et al., 2012; Rawdon et al., 2018), have also reported modest changes in symptoms. Tentatively, the present finding suggests that the benefits of EBT may be greater for young people. This could be due to age-related differences in emotional face processing, as previous studies have shown that neural activation of the amygdala (a brain area known to be affected by successful EBT; Penton-Voak et al., 2020) plays a larger role in emotional face processing in younger, rather than older, adults (Fischer, Nyberg, & Bäckman, 2010). Alternatively, these age effects may be due to symptom duration, with older participants potentially have experienced longer periods of poor mental health. These unplanned analyses require further investigation and should be treated as hypothesis-generating. If this pattern is confirmed, it may have important theoretical implications, indicating that disruptions in emotional processing are more central to the development of mental health symptoms in young people.

One potential explanation for the small effects found across these studies is that our measure of emotion processing (i.e. emotional balance point) may suffer from poor validity. Baseline correlation analyses suggested that emotional balance point was associated with several measures of mental health in both studies. However, these correlations were modest, and the average emotional balance point was similar in both study populations – despite one population consisting of healthy participants and one consisting of participants receiving treatment for low mood. While such comparisons require formal testing in a future case−control study, this data points towards our measure of emotion processing not being strongly tied to participants’ mood, limiting the capacity that EBT has to produce large transfer effects. Further research is required to determine whether these modest associations (and subsequent transfer effects) are due to using a measure with poor validity, or whether the causal association between emotional processing biases and mental health is overstated.

Study 2 has important limitations. Firstly, we lacked control over SSRI duration or type. We adopted a pragmatic recruitment strategy in order to test EBT in a larger sample of participants. This relied on participants self-reporting their SSRI medication and treatment duration. Participants reported a range of SSRI types and significant variation in the length of their use (mean 50 months). The extent of SSRI-associated neurogenesis at these durations is unknown and previous studies suggest SSRIs can alter emotion perception after only a few doses (Godlewska et al., 2016). Therefore, participants may have already maximally benefitted from such changes earlier in their medication course. Secondly, as study 2 was insufficiently powered, the findings should be regarded as preliminary. Future research should investigate EBT as an adjunct therapy in participants who are beginning SSRI treatment, potentially controlling for SSRI type (the effects of which are currently understudied). Administering EBT at this early stage may allow for quicker, or more pronounced, changes in emotional perception and hasten any resulting clinical benefit. Longer time periods should also be explored, as previous research suggests that benefits following changes in emotional bias may appear several weeks later (Harmer et al., 2009). Our investigation demonstrates that patients with emotional disorders can readily incorporate a remote digital therapeutic into their treatment routine.

Despite minimal changes in symptoms, participants receiving active EBT reported that they felt more positively about their current treatment for low mood than participants receiving sham EBT. This effect remained at follow-up. As this was not a primary outcome of the investigation, we did not collect sufficient qualitative data to determine the nature of this change. It is unclear whether this is the result of an EBT-induced positivity bias, or a by-product of engaging with a mildly challenging cognitive task. This warrants further study, as beliefs surrounding treatment can influence adherence to antidepressant medication (e.g. Brown et al., 2005). A brief digital intervention may promote more positive health behaviours by improving engagement with treatment.

Taken together, these studies demonstrate the feasibility of delivering a multi-session digital intervention to promote positive changes in emotion perception in healthy and medicated samples. However, evidence of transfer effects was weak and inconsistent, suggesting EBT does not currently serve as a therapeutic and requires development before being deployed as a treatment for emotional disorders.

## Supplementary Material

Supplementary material. The supplementary material for this article can be found at https://doi.org/10.1017/S0033291721002014.

Supplementary Materials

## Figures and Tables

**Table 1:** Baseline characteristics (Means and Standard Deviations) of study samples

Baseline measure	Study 1: Healthy participants		Study 2: Participants on medication	
	Sham (*n* = 251)	Active (*n* = 271)	Sham (*n* = 104)	Active (*n* = 108)
Age (years)	34.8 (11.7)	34.1 (10.5)	37.2 (11.6)	37.0 (10.7)
Emotional bias (balance point)	7.7 (1.4)	7.8 (1.2)	7.7 (1.3)	7.6 (1.4)
Daily stress (DSI-AIR)	2.59 (0.86)	2.48 (0.86)	3.63 (1.07)	3.65 (0.98)
Effort for reward (EEfRT)	0.45 (0.28)	0.41 (0.26)	–	–
Anhedonia (SHAPS)	–	–	3.38 (3.26)	3.36 (2.96)
Quality of life (QLES)	62.66 (14.41)	64.28 (13.74)	49.78 (16.01)	48.71 (15.52)
Depressive symptoms (PHQ-9)	6.53 (4.74)	6.37 (4.91)	11.63 (5.69)	12.22 (5.22)
State anxiety (STAI-Y1)	40.77 (12.44)	40.29 (12.52)	52.73 (13.27)	52.60 (12.67)
Trait anxiety (STAI-Y2)	45.08 (12.25)	44.51 (12.70)	56.69 (11.34)	57.89 (10.59)
SSRI duration (months)	–	–	46.9 (54.6)	52.0 (61.0)
Feeling of treatment	–	–	1.69 (0.76)	1.34 (0.99)

*Note.* DSI-AIR: Daily Stress Inventory (Average Impact Rating), EEfRT: Effort Expenditure for Rewards Task, SHAPS: Snaith-Hamilton Pleasure Scale, QLES: Quality of Life Enjoyment and Satisfaction Questionnaire (short form), PHQ-9: Patient Health Questionnaire, STAI: State Trait Anxiety Inventory, SSRI: Selective Serotonin Reuptake Inhibitor. Possible score ranges: DSI-AIR (1-7), EEfRT (0-1), SHAPS (0-14), QLES (0-100), PHQ-9 (0-24), STAI-Y1 (20-80), STAI-Y2 (20-80), Feeling of Treatment (−2 to +2).

**Table 2:** Study 1 effects of training condition on mood and cognitive outcomes

Outcome	Model 1			Model 2		
	*b*	95% CI	*p*	*b*	95% CI	*p*
Post-training (Session 4)						
Emotional bias (Balance point)	2.6	2.3–2.9	<0.001	2.6	2.3–2.9	<0.001
Daily stress (DSI-AIR)	−0.04	−0.15 to 0.08	0.518	−0.04	−0.15 to 0.07	0.500
Effort for reward (EEfRT)	0.01	−0.03 to 0.05	0.705	0.01	−0.03 to 0.05	0.678
Quality of life (QLES)	0.58	−1.09 to 2.25	0.494	0.58	−1.05 to 2.20	0.486
Follow-up (Session 5)						
Emotional bias (Balance point)	2.0	1.7–2.3	<0.001	2.0	1.7–2.3	<0.001
Daily stress (DSI-AIR)	−0.04	−0.17 to 0.09	0.555	−0.04	−0.17 to 0.08	0.524
Effort for reward (EEfRT)	0.03	−0.02 to 0.07	0.225	0.03	−0.02 to 0.07	0.219
Quality of life (QLES)	−1.32	−3.14 to 0.49	0.152	−1.33	−3.10 to 0.44	0.140

*Note*. Model 1: Adjusted for baseline (session 1) measure of the respective outcome and basic demographics (age and sex). Model 2: Additionally adjusted for baseline mental health: state anxiety (STAI-Y1) and depressive symptoms (PHQ-9).

**Table 3:** Study 1 interaction of training condition and baseline mental health on post-training outcomes

Moderator	Model	Emotional bias (BP)			Daily stress (DSI-AIR)			Effort for reward (EEfRT)			Quality of life (QLES)		
		*n*	*b* (95% CI)	*p*	*n*	*b* (95% CI)	*p*	*n*	*b* (95% CI)	*p*	*n*	*b* (95% CI)	*p*
Depressive symptoms(PHQ-9)	*Stratum specific*												
	No symptoms	225	2.9 (2.5–3.4)	<0.001	225	0.05 (−0.11 to 0.20)	0.545	225	0.01 (−0.05 to 0.08)	0.629	225	−1.10 (−3.04 to 0.84)	0.265
	Symptomatic	297	2.3 (2.0–2.7)	<0.001	297	−0.09 (−0.25 to 0.07)	0.274	297	−0.01 (−0.07 to 0.05)	0.731	297	2.36 (−0.11 to 4.83)	0.061
	Interaction	522	−0.6 (−1.1 to −0.0)	0.033	522	−0.15 (−0.38 to 0.08)	0.208	522	−0.02 (−0.11 to 0.06)	0.591	522	3.59 (0.27–6.85)	0.034
Trait anxiety (STAI-Y2)	*Stratum specific*												
	Low	271	2.9 (2.6–3.3)	<0.001	271	0.00 (−0.15 to 0.15)	0.986	271	0.01 (−0.05 to 0.07)	0.764	271	0.24 (−1.82 to 2.29)	0.820
	High	251	2.2 (1.8–2.6)	<0.001	251	−0.05 (−0.23 to 0.12)	0.543	251	−0.01 (−0.07 to 0.05)	0.773	251	1.35 (−1.24 to 3.95)	0.306
	Interaction	522	−0.7 (−1.3 to −0.2)	0.006	522	−0.06 (−0.29 to 0.17)	0.587	522	−0.02 (−0.10 to 0.06)	0.671	522	1.20 (−2.08 to 4.47)	0.473

*Note.* BP: Balance Point; DSI-AIR: Daily Stress Inventory (Average Impact Rating); EEfRT: Effort Expenditure for Rewards Task; QLES: Quality of Life Enjoyment and Satisfaction Questionnaire (short form); PHQ-9: Patient Health Questionnaire; STAI: State-Trait Anxiety Inventory.

**Table 4:** Study 2 effects of training condition on mood and cognitive outcomes

Outcome	Model 1			Model 2		
	*b*	95% CI	*p*	*b*	95% CI	*p*
Post-training (Session 4)						
Emotional bias (Balance point)	2.3	1.9–2.7	<0.001	2.3	1.9–2.7	<0.001
Quality of life (QLES)	1.61	−1.03 to 4.25	0.231	1.47	−1.18 to 4.13	0.276
Daily stress (DSI-AIR)	−0.05	−0.25 to 0.15	0.614	−0.06	−0.26 to 0.14	0.578
State anxiety (STAI-Y1)	−1.36	−3.45 to 0.73	0.201	−1.46	−3.57 to 0.64	0.172
Anhedonia (SHAPS)	−0.36	−1.09 to 0.37	0.330	−0.41	−1.14 to 0.32	0.268
Depressive symptoms (PHQ-9)	−0.31	−1.16 to 0.54	0.472	−0.32	−1.17 to 0.54	0.463
Feeling of treatment	0.36	0.12–0.59	0.003	0.35	0.11–0.59	0.004
Follow-up (Session 5)						
Emotional bias (Balance point)	1.8	1.4–2.2	<0.001	1.8	1.4–2.2	<0.001
Quality of life (QLES)	2.59	−0.36 to 5.54	0.085	2.29	−0.64 to 5.23	0.125
Daily stress (DSI-AIR)	−0.10	−0.32 to 0.12	0.364	−0.10	−0.32 to 0.12	0.369
State anxiety (STAI-Y1)	−1.18	−3.59 to 1.24	0.339	−1.22	−3.66 to 1.22	0.326
Anhedonia (SHAPS)	−0.39	−1.21 to 0.43	0.347	−0.43	−1.25 to 0.40	0.308
Depressive symptoms (PHQ-9)	−0.30	−1.31 to 0.72	0.563	−0.26	−1.27 to 0.76	0.622
Feeling of treatment	0.26	0.04–0.48	0.021	0.27	0.05–0.48	0.018

*Note.* Model 1: Adjusted for baseline (session 1) measure of the respective outcome and basic demographics (age and sex). Model 2: Additionally adjusted for length of SSRI use and additional therapy use.

## Data Availability

The datasets underlying the results reported here, and accompanying R analysis scripts, are available on the University of Bristol data repository, data.bris, at https://doi.org/10.5523/bris.lowf35tsgxav2rnb1jpvjvkm3 and https://doi.org/10.5523/bris.1jk6puznegx4121cjt72j1mln9.
