# Supplementary material for: Emotional bias training as a treatment for anxiety and depression: evidence from experimental medicine studies in healthy and medicated samples
Source: Psychol Med. Author manuscript; Available in PMC 2023 Mar 3. (PMC9975995; doi:10.1017/S0033291721002014)
Supplement: Supplementary Materials [file EMS153902-supplement-Supplementary_Materials.pdf]

SUPPLEMENTARY MATERIAL

Supplementary Tables

Table S1. Study 1 Interaction of Training Condition and Baseline Mental Health on Outcomes at Follow-Up (Session 5)

		Emotional Bias (BP)			Daily Stress (DSI-AIR)			Effort for Reward (EEfRT)			Quality of Life (QLES)		
Moderator	Model	*n*	*b* [95% CI]	*p*	*n*	*b* [95% CI]	*p*	*n*	*b* [95% CI]	*p*	*n*	*b* [95% CI]	*p*
Depressive Symptoms (PHQ-9)	*Stratum specific*												
	No Symptoms	225	2.3 [1.9, 2.7]	<.001	225	-0.05 [-0.23, 0.12]	.539	225	0.00 [-0.06, 0.07]	.930	225	-0.85 [-3.33, 1.64]	.502
	Symptomatic	297	1.8 [1.4, 2.2]	<.001	297	0.01 [-0.17, 0.19]	.888	297	0.02 [-0.04, 0.08]	.456	297	-0.90 [-3.42, 1.62]	.483
	Interaction	522	-0.5 [-1.1, 0.1]	.082	522	0.06 [-0.19, 0.32]	.629	522	0.02 [-0.06, 0.11]	.622	522	-0.04 [-3.65, 3.55]	.979
Trait Anxiety (STAI-Y2)	*Stratum specific*												
	Low	271	2.2 [1.9, 2.6]	<.001	271	-0.09 [-0.25, 0.07]	.265	271	0.01 [-0.05, 0.07]	.767	271	-0.08 [-2.50, 2.35]	.951
	High	251	1.7 [1.3, 2.1]	<.001	251	0.07 [-0.13, 0.27]	.482	251	0.02 [-0.04, 0.08]	.555	251	-2.02 [-4.66, 0.61]	.131
	Interaction	522	-0.6 [-1.1, -0.0]	.043	522	0.16 [-0.09, 0.41]	.217	522	0.01 [-0.07, 0.10]	.748	522	-1.78 [-5.35, 1.79]	.327

Note. BP: Balance Point, DSI-AIR: Daily Stress Inventory (Average Impact Rating), EEfRT: Effort Expenditure for Rewards Task, QLES: Quality of Life Enjoyment and Satisfaction Questionnaire (short form), PHQ-9: Patient Health Questionnaire, STAI: State Trait Anxiety Inventory. Models adjusted for baseline measure of outcome.

Table S2. Study 2 Interaction of Training Condition and Baseline Mental Health on Post-training Outcomes (Session 4)

			Quality of life (QLES)		Daily Stress (DSI-AIR)		Anhedonia (SHAPS)		Feeling of Treatment	
Moderator	Model	*n*	*b* [95% CI]	*p*	*b* [95% CI]	*p*	*b* [95% CI]	*p*	*b* [95% CI]	*p*
Depressive Symptoms (PHQ-9)	*Stratum specific*									
	Low	112	1.62 [-1.59, 4.83]	.320	0.02 [-0.23, 0.28]	.852	-0.21 [-0.97, 0.55]	.585	0.40 [0.11, 0.70]	.007
	High	100	1.98 [-2.30, 6.27]	.360	-0.16 [-0.48, 0.16]	.313	-0.61 [-1.90, 0.69]	.353	0.37 [-0.01, 0.76]	.058
	Interaction	212	0.33 [-4.94, 5.61]	.901	-0.18 [-0.58, 0.22]	.364	-0.36 [-1.81, 1.10]	.631	0.07 [-0.40, 0.54]	.775
Trait Anxiety (STAI-Y2)	*Stratum specific*									
	Low	106	1.65 [-1.71, 5.00]	.333	0.03 [-0.24, 0.30]	.830	-0.58 [-1.31, 0.15]	.118	0.29 [-0.01, 0.59]	.062
	High	106	3.27 [-0.85, 7.39]	.118	-0.23 [-0.53, 0.07]	.133	-0.73 [-1.96, 0.50]	.242	0.52 [0.13, 0.91]	.009
	Interaction	212	1.44 [-3.81, 6.68]	.590	-0.27 [-0.67, 0.13]	.184	-0.24 [-1.65, 1.18]	.742	0.24 [-0.24, 0.72]	.318

Note. QLES: Quality of Life Enjoyment and Satisfaction Questionnaire (short form), DSI-AIR: Daily Stress Inventory (Average Impact Rating), SHAPS: Snaith-Hamilton Pleasure Scale, PHQ-9: Patient Health Questionnaire, STAI: State Trait Anxiety Inventory.

Table S3. Study 2 Interaction of Training Condition and Baseline Mental Health on Outcomes at Follow-Up (Session 5)

			Quality of Life (QLES)		Daily Stress (DSI-AIR)		Anhedonia (SHAPS)		Feeling of Treatment	
Moderator	Model	*n*	*b* [95% CI]	*p*	*b* [95% CI]	*p*	*b* [95% CI]	*p*	*b* [95% CI]	*p*
Depressive Symptoms (PHQ-9)	*Stratum specific*									
	Low	112	1.15 [-2.23, 4.53]	.502	-0.01 [-0.28, 0.27]	.953	-0.41 [-1.27, 0.45]	.349	0.41 [0.10, 0.72]	.010
	High	100	4.49 [-0.49, 9.46]	.077	-0.24 [-0.59, 0.11]	.174	-0.41 [-1.86, 1.05]	.580	0.14 [-0.16, 0.43]	.352
	Interaction	212	3.31 [-2.57, 9.19]	.268	-0.23 [-0.66, 0.21]	.313	0.04 [-1.59, 1.68]	.957	-0.18 [-0.60, 0.25]	.410
Trait Anxiety (STAI-Y2)	*Stratum specific*									
	Low	106	2.17 [-1.52, 5.87]	.246	-0.12 [-0.39, 0.15]	.381	-0.90 [-1.66, -0.13]	.022	0.29 [-0.01, 0.59]	.057
	High	106	4.70 [0.04, 9.35]	.048	-0.22 [-0.58, 0.14]	.227	-0.40 [-1.85, 1.05]	.583	0.37 [0.07, 0.66]	.017
	Interaction	212	2.35 [-3.51, 8.22]	.430	-0.09 [-0.54, 0.35]	.675	0.45 [-1.16, 2.07]	.582	0.09 [-0.33, 0.51]	.676

Note. QLES: Quality of Life Enjoyment and Satisfaction Questionnaire (short form), DSI-AIR: Daily Stress Inventory (Average Impact Rating), SHAPS: Snaith-Hamilton Pleasure Scale, PHQ-9: Patient Health Questionnaire, STAI: State Trait Anxiety Inventory. Models adjusted for baseline measure of outcome.

Table S4. Study 2 Interaction of Training Condition and Age on Mood and Cognitive Outcomes

		Younger Stratum (*n* = 107)		Older Stratum (*n* = 105)		Interaction (*n* = 212)	
Time	Outcome	*b* [95% CI]	*p*	*b* [95% CI]	*p*	*b* [95% CI]	*p*
Post-training (Session 4)	Emotional Bias (BP)	2.3 [1.7, 2.9]	<.001	2.4 [1.7, 3.0]	<.001	0.1 [-0.8, 0.9]	.872
	Quality of Life (QLES)	4.54 [0.91, 8.17]	.015	-1.38 [-5.22, 2.46]	.478	-5.86 [-11.07, -0.64]	.028
	Daily Stress (DSI-AIR)	-0.19 [-0.47, 0.08]	.163	0.09 [-0.21, 0.39]	.569	0.28 [-0.12, 0.68]	.174
	Anhedonia (SHAPS)	-0.67 [-1.68, 0.35]	.195	-0.13 [-1.20, 0.95]	.815	0.51 [-0.95, 1.97]	.491
	State Anxiety (STAI-Y1)	-5.13 [-8.07, -2.20]	.001	2.28 [-0.60, 5.16]	.120	7.31 [3.25, 11.38]	<.001
	Depression (PHQ-9)	-1.17 [-2.24, -0.09]	.034	0.52 [-0.81, 1.86]	.440	1.60 [-0.09, 3.29]	.063
	Feeling of Treatment	0.35 [0.00, 0.70]	.047	0.35 [0.01, 0.68]	.041	-0.02 [-0.49, 0.45]	.933
Follow-up (Session 5)	Emotional Bias (BP)	1.9 [1.3, 2.4]	<.001	1.9 [1.3, 2.5]	<.001	-0.0 [-0.8, 0.8]	.962
	Quality of Life (QLES)	6.17 [2.10, 10.24]	.003	-0.83 [-5.00, 3.33]	.692	-6.62 [-12.43, -0.80]	.026
	Daily Stress (DSI-AIR)	-0.28 [-0.58, 0.03]	.077	0.05 [-0.26, 0.37]	.736	0.32 [-0.13, 0.76]	.159
	Anhedonia (SHAPS)	-0.87 [-2.01, 0.27]	.132	0.09 [-1.13, 1.30]	.889	0.90 [-0.75, 2.54]	.283
	State Anxiety (STAI-Y1)	-4.19 [-8.03, -0.35]	.033	1.79 [-1.08, 4.67]	.219	5.74 [0.95, 10.53]	.019
	Depression (PHQ-9)	-0.79 [-2.28, 0.69]	.292	0.11 [-1.29, 1.51]	.873	0.83 [-1.19, 2.86]	.418
	Feeling of Treatment	0.45 [0.16, 0.73]	.002	0.13 [-0.21, 0.47]	.438	-0.22 [-0.65, 0.21]	.313

Note. BP: Balance Point, QLES: Quality of Life Enjoyment and Satisfaction Questionnaire (short form), DSI-AIR: Daily Stress Inventory (Average Impact Rating), SHAPS: Snaith-Hamilton Pleasure Scale, STAI: State Trait Anxiety Inventory, PHQ-9: Patient Health Questionnaire. Younger and older strata created by median split (older > 35 years). All models adjusted for baseline measure of outcome and participant sex.

Table S5. Study 1 Interaction of Training Condition and Age on Study Outcomes

			Emotional Bias (BP)		Daily Stress (DSI-AIR)		Effort for Reward (EEfRT)		Quality of Life (QLES)	
Time	Model	*n*	*b* [95% CI]	*p*	*b* [95% CI]	*p*	*b* [95% CI]	*p*	*b* [95% CI]	*p*
Post-Training (Session 4)	*Stratum specific*									
	Younger	268	2.8 [2.4, 3.2]	<.001	-0.12 [-0.28, 0.05]	.160	0.04 [-0.02, 0.10]	.201	0.51 [-1.88, 2.90]	.676
	Older	254	2.4 [2.0, 2.8]	<.001	0.06 [-0.11, 0.22]	.506	-0.02 [-0.07, 0.03]	.424	0.52 [-1.82, 2.85]	.662
	Interaction	522	-0.3 [-0.9, 0.2]	.214	0.16 [-0.06, 0.39]	.160	-0.05 [-0.13, 0.03]	.217	-0.25 [-3.54, 3.03]	.878
Follow-Up (Session 5)	*Stratum specific*									
	Younger	268	2.1 [1.7, 2.4]	<.001	-0.12 [-0.31, 0.08]	.238	0.04 [-0.02, 0.11]	.187	-1.77 [-4.34, 0.80]	.177
	Older	254	2.0 [1.6, 2.3]	<.001	0.07 [-0.10, 0.23]	.441	0.01 [-0.04, 0.07]	.655	-1.03 [-3.59, 1.52]	.426
	Interaction	522	-0.0 [-0.6, 0.5]	.925	0.16 [-0.09, 0.41]	.211	-0.02 [-0.11, 0.06]	.606	0.12 [-3.45, 3.69]	.947

Note. BP: Balance Point, DSI-AIR: Daily Stress Inventory (Average Impact Rating), EEfRT: Effort Expenditure for Rewards Task, QLES: Quality of Life Enjoyment and Satisfaction Questionnaire (short form). Younger and older strata created by median split (older > 32 years). All models adjusted for baseline measure of outcome and participant sex.

Table S6. Correlations between emotional bias (balance point) and study outcome measures at baseline (session 1, pre-training)

	Study 1 (n = 522)		Study 2 (n = 212)	
	r	p	r	p
Depressive Symptoms (PHQ-9)	-0.04	.329	-0.15	.027
State Anxiety (STAI-Y1)	-0.05	.248	-0.10	.130
Trait Anxiety (STAI-Y2)	-0.10	.029	-0.12	.082
Daily Stress (DSI-AIR)	-0.10	.022	-0.07	.312
Quality of Life (QLES)	0.09	.034	0.16	.022
Anhedonia (SHAPS)	-	-	-0.21	.002
Effort for Reward (EEfRT)	0.00	.974	-	-
Feeling of Treatment	-	-	0.00	.973

Note. PHQ-9: Patient Health Questionnaire, STAI: State Trait Anxiety Inventory, QLES: Quality of Life Enjoyment and Satisfaction Questionnaire (short form), DSI-AIR: Daily Stress Inventory (Average Impact Rating), SHAPS: Snaith-Hamilton Pleasure Scale, EEfRT: Effort Expenditure for Rewards Task.

Supplementary Methods

Figure S1. Schematic of a training block trial in the emotional bias training task.

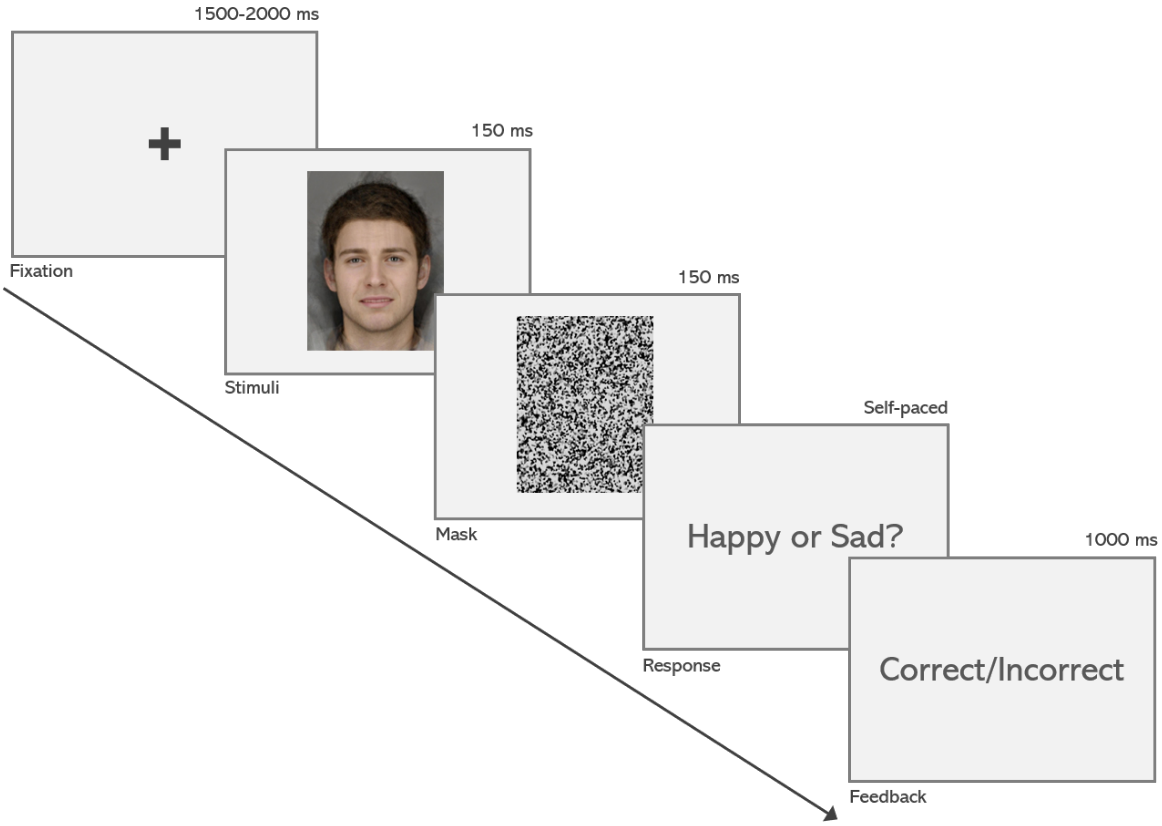

An example trial during the training (second) block of an EBT session. Participants respond to a series of emotional faces, categorising each as “happy” or “sad”, and receive immediate feedback as to whether this was “Correct” or “Incorrect”. In the active condition, this feedback is tailored to train the participant to perceive an additional 2 faces as “happy” in comparison to their responses in the baseline block. In the sham condition, this feedback is matched to their baseline responses (i.e. no training is administered).

Figure S2. Consort diagram for Study 1 Sample (Healthy Participants)

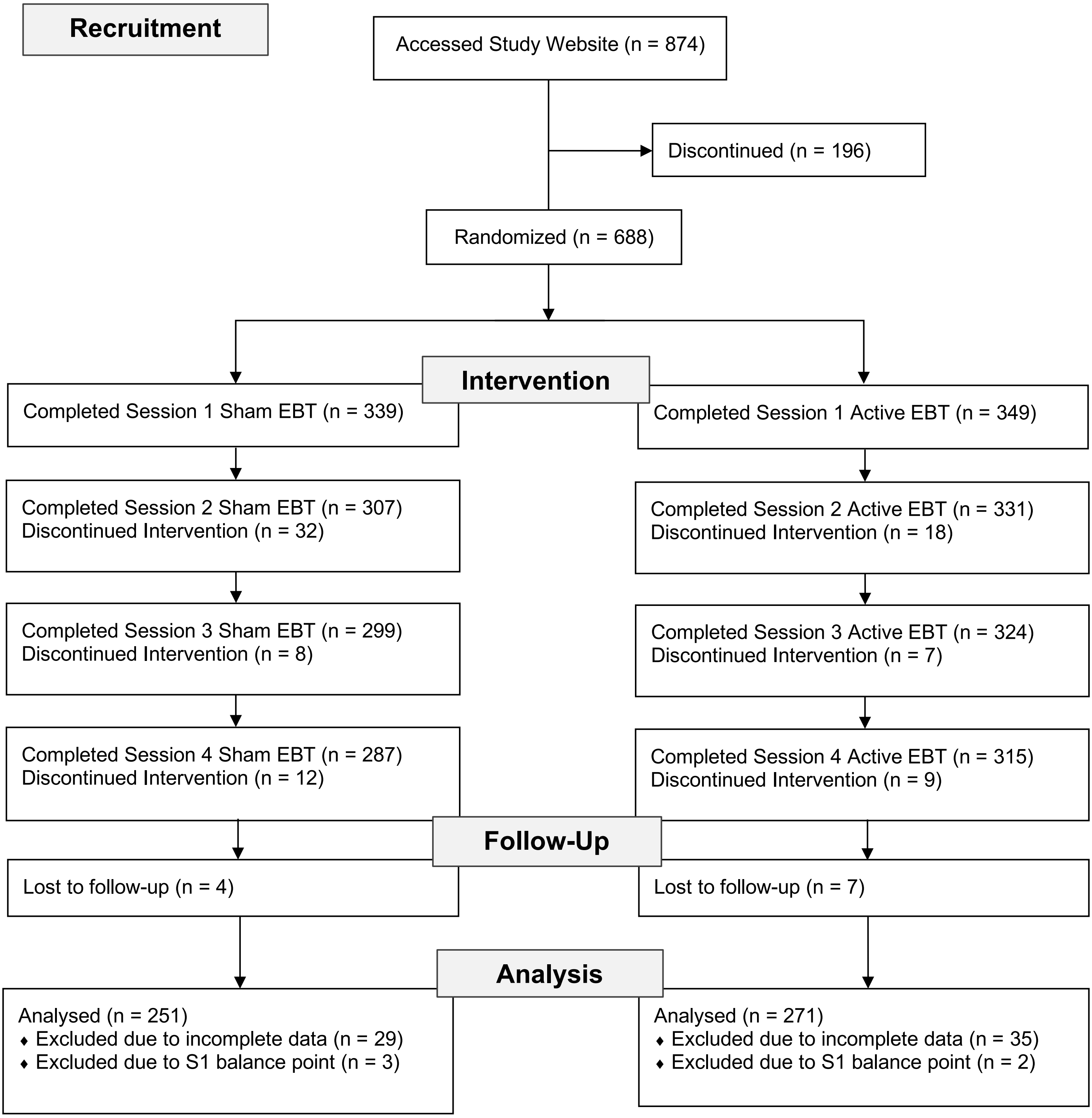

Figure S3. Consort diagram for Study 2 Sample (Participants taking SSRIs)

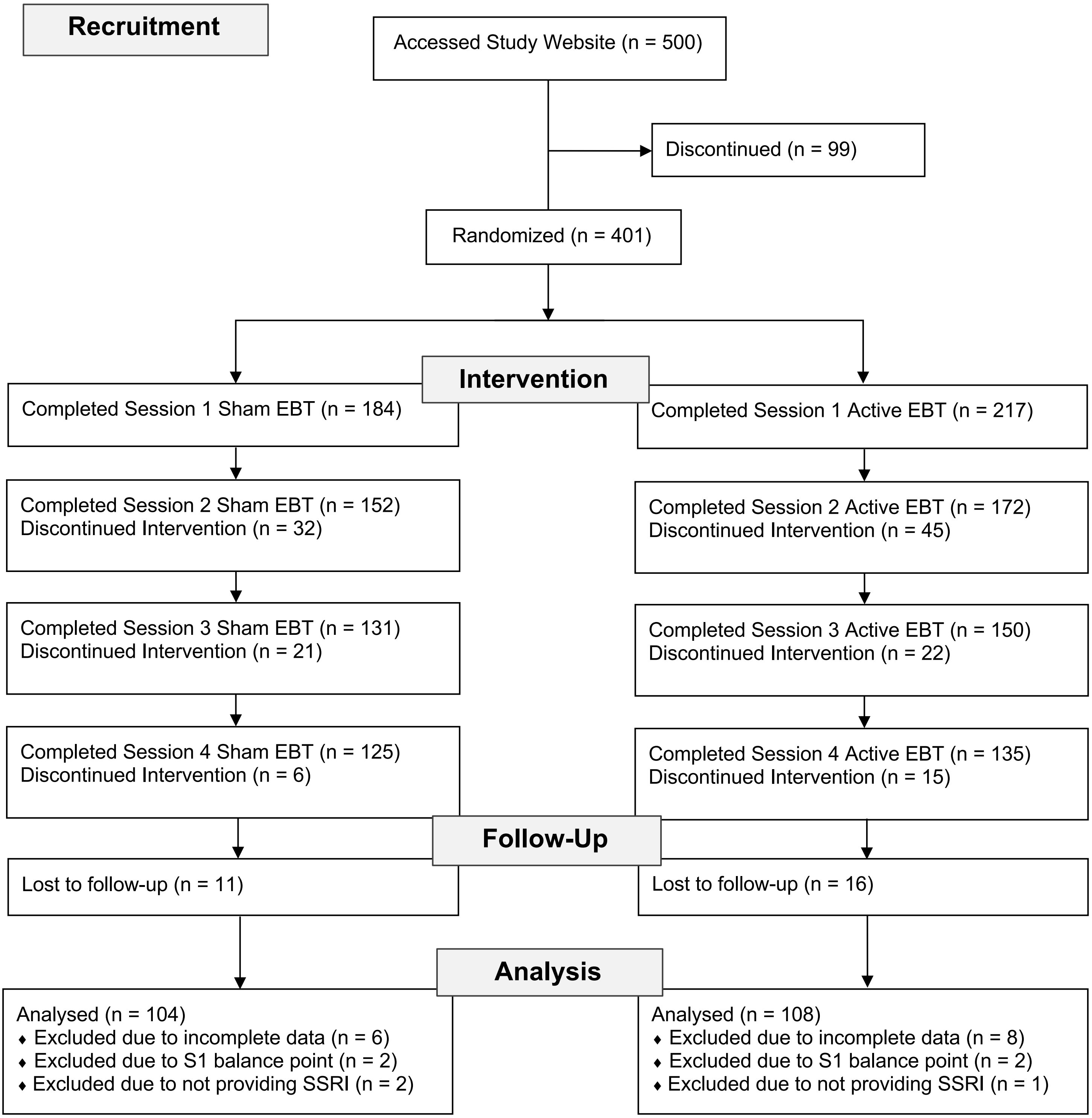

Effort Expenditure for Reward Task (EEfRT)

The EEfRT is designed as an objective cognitive measure of anhedonia that measures the amount of physical effort an individual is willing to expend in order to earn a reward (Treadway, Buckholtz, Schwartzman, Lambert, & Zald, 2009). Participants were given a choice between ‘easy’ and ‘hard’ trials in return for different monetary rewards. The easy trials required the participant to tap the spacebar repeatedly, while the hard trials required participants to press four keys in sequence: ‘Q’, ‘C’, ‘M’ and ‘P’. We adapted the ‘hard’ trials from the original task (which required participants to press the space bar with their little finger) to account for the present study being conducted remotely, and therefore participants adherence to task instructions could not be monitored (i.e., participants could evade hard trials by using their index finger).

The number of presses required to fill the bar depended on the participant's performance in a brief calibration phase (framed as a practice phase), which took place before the task began. In this phase, participants had to attempt 4 trials, each requiring them to ‘fill a bar’ that requires a different number of button presses within 21 seconds, using the hard trial button sequence. We recorded the average number of button presses per second in the second and third of these trials, and used this to determine the maximum possible number of presses the participant could have achieve within 21 seconds. Based on pilot experiments, we then increased this value by 15%, thus ensuring that the hard trials were truly difficult and required significant effort to complete. This value was then used as the required number of presses in the following hard trials, with the caveat that we set the maximum required presses at 300, and the minimum at 50. Practice trial four also required this many button presses, to give the participant an indication as to the difficulty of the trials they would face. In contrast, easy trials required 15% of the hard trial button presses, with a minimum of 20 and a maximum of 30.

A trial was considered successful when a participant filled up the bar on the screen to the red line by repeatedly pressing buttons, however this did not guarantee the participant a reward. Hard trials were always worth more than easy trials (15p), but both types of trial came with a shared probability cue (12%, 50%, or 88%), which indicated the likelihood of receiving a reward if the participant successfully fills the bar to the red line (Figure S4). Upon filling the bar, the participant was presented with a spinner; if the arrow landed in the green segment, the reward in the trial was won. If participants won at least 5 trials, two of the rewards from these trials were selected at random as real ‘bonus money’ to their study compensation.

Figure S4. Example Trial of the Effort-Expenditure for Rewards Task

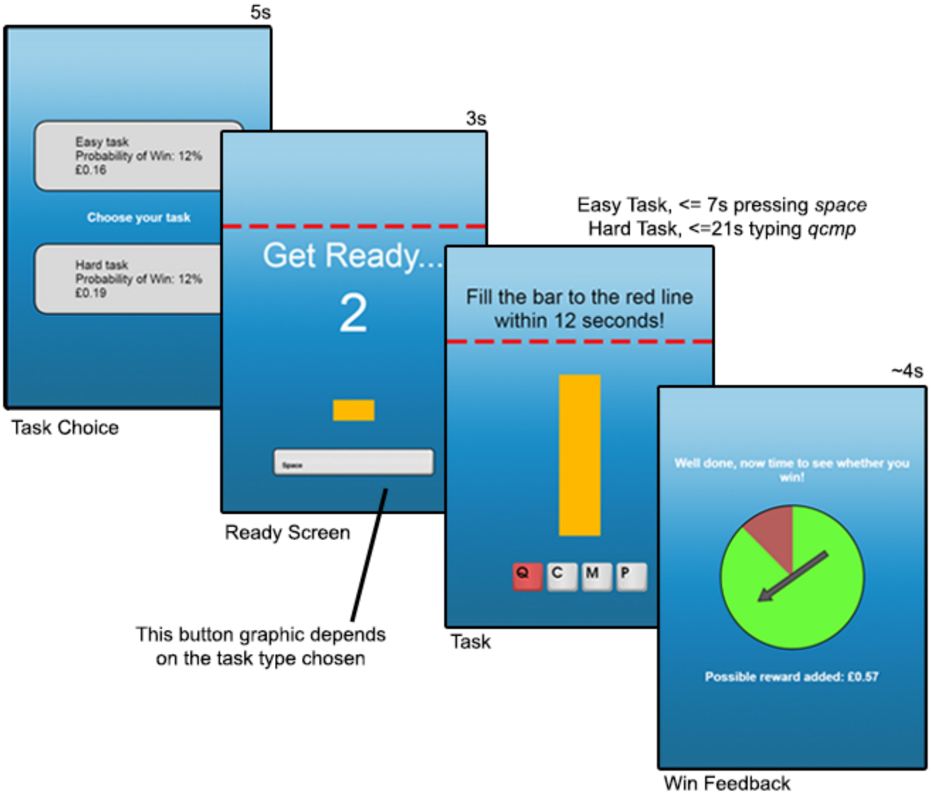

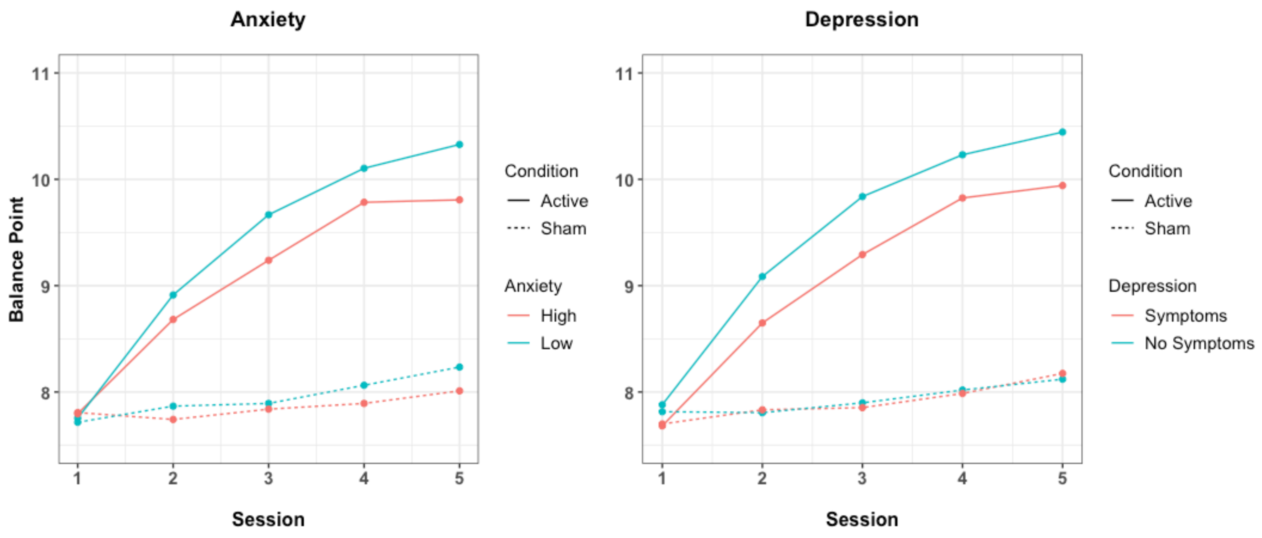

Figure S5. Study 1 (healthy participants) mean emotional balance point at each session, stratified by EBT condition and mental health symptoms. Means are from the baseline (pre-training) block of that EBT session. Sessions 1-4 were completed within a 10-day period. Session 5 was completed approximately 2 weeks after Session 4. **Left:** Participants are stratified by high and low trait anxiety (high: STAI-Y2 >44). **Right:** Participants are stratified by depressive symptoms (with symptoms: PHQ-9 >4).
